# Principles of open, transparent and reproducible science in author guidelines of sleep research and chronobiology journals

**DOI:** 10.12688/wellcomeopenres.16111.2

**Published:** 2021-02-26

**Authors:** Manuel Spitschan, Marlene H. Schmidt, Christine Blume

**Affiliations:** 1 Department of Experimental Psychology, University of Oxford, Oxford, UK; 2 Centre for Chronobiology, Psychiatric Hospital of the University of Basel (UPK), Basel, Switzerland; 3 Transfaculty Research Platform Molecular and Cognitive Neurosciences, University of Basel, Basel, Switzerland

**Keywords:** sleep, chronobiology, circadian rhythms, publishing, open science, meta research

## Abstract

**Background: **"Open science" is an umbrella term describing various aspects of transparent and open science practices. The adoption of practices at different levels of the scientific process (e.g., individual researchers, laboratories, institutions) has been rapidly changing the scientific research landscape in the past years, but their uptake differs from discipline to discipline. Here, we asked to what extent journals in the field of sleep research and chronobiology encourage or even require following transparent and open science principles in their author guidelines.

**Methods: **We scored the author guidelines of a comprehensive set of 27 sleep and chronobiology journals, including the major outlets in the field, using the standardised Transparency and Openness (TOP) Factor. The TOP Factor is a quantitative summary of the extent to which journals encourage or require following various aspects of open science, including data citation, data transparency, analysis code transparency, materials transparency, design and analysis guidelines, study pre-registration, analysis plan pre-registration, replication, registered reports, and the use of open science badges.

**Results: **Across the 27 journals, we find low values on the TOP Factor (median [25 ^th^, 75 ^th^ percentile] 3 [1, 3], min. 0, max. 9, out of a total possible score of 29) in sleep research and chronobiology journals.

**Conclusions: **Our findings suggest an opportunity for sleep research and chronobiology journals to further support recent developments in transparent and open science by implementing transparency and openness principles in their author guidelines.

## Introduction

During the past few years, the open science movement gained increasing popularity and is rapidly changing the way science is done, especially among early career researchers striving to improve scientific practice and overcome deficits in the current scientific *status quo* ^1, 2^. The term “open science” is relatively ill-defined and includes a range of different methods, tools, platforms, and practices that are geared to improving the quality of science through transparency ^3^. At present, it is still largely up to individual researchers and research groups to decide to what extent they want to engage in open science practices and incentives that may promote open science are rare. Journals as the main outlets for archival scientific dissemination can support the movement and offer ways to make the scientific process more open, reproducible, and emphasise good scientific practice. They may even speed up the process by requiring authors to adhere to open science standards. However, to what extent do journals in the fields of sleep and chronobiology encourage or even require following the standards of open science?

The scientific fields of sleep research and chronobiology concern all aspects of sleep and circadian rhythmicity. As almost all aspects of physiology and behaviour are under some type of circadian control, this cluster of scientific fields is fundamentally interdisciplinary, employing a wide variety of methodologies. Therefore, this research area is very heterogeneous, drawing from different ‘core’ disciplines (including neuroscience, psychology, molecular biology, and others), each with their own scientific history, and the degree to which open science principles are adopted may vary widely.

In this study, we asked to what extent scientific journals specialised on sleep research and chronobiology lay out open-science principles in their author guidelines. Inspired by previous publications in the field of pain research ^4, 5^, we assessed the implementation of research transparency and openness in journal guidelines using the quantitative Transparency and Openness Factor ^6^ (TOP Factor). The TOP Factor, launched in February 2020, is based on the TOP guidelines and provides a normative standard for research transparency and openness. It contains ten sub-scales, corresponding to different aspects of openness and transparency in scientific research, reflecting the *Transparency and Openness Promotion Guidelines*: data citation, data transparency, analysis code transparency, materials transparency, design and analysis guidelines, study pre-registration, analysis plan pre-registration, replication, registered reports, and open science badges.

The TOP Factor recognises different levels relating to mentioning, encouraging, requiring and enforcing specific transparency and openness practices, which are implemented in a verbally anchored rating scheme. The specific practices are:

- *Data citation* refers to the citation of data in a repository using standard means, including a digital object identifier (DOI). Citing the data set(s) that is/are reported on in a given publication facilitates access to these data, independent re-analyses by other authors times, and validation of results, thereby likely increasing the probability that a given result can be reproduced by a different group, or at a later time.
- *Data, analysis code,* and *materials transparency* refers to making data, analysis code and materials available as part of the journal submission. The availability of original data again supports the reproducibility of a given reported research result, as other investigators will be able to analyse the data independently. Furthermore, the availability of data also supports meta-analysis or evidence synthesis efforts. The availability of code is a necessary step to be able to reproduce the exact same analysis again at a later time. Finally, availability of materials used in the study ensures that other investigators can independently replicate the research design and expand on it in a way that is consistent with the original publication.
- *Design and analysis guidelines* refers to the inclusion of instruments describing the study design and analysis formally, such as the Preferred Reporting Items for Systematic Reviews and Meta-Analyses (PRISMA) or Consolidated Standards of Reporting Trials (CONSORT) standards. These standard methods aid in providing an overview of the methodological rigour of the study and facilitate appraisal of completeness of a given research study.
- *Study pre-registration* and *analysis pre-registration* refers to the pre-registration of data collection and/or analysis prior to their execution. Pre-registering study designs and analysis can be a countermeasure against HARKing (hypothesizing after the results are known) ^7^ and other questionable research practices (QRPs) ^8^. Pre-registration can take many forms, and most often refers to specifying the research design in advance of data collection, as well as the data analysis plan, so as to avoid “fishing” for positive results.
- *Replication* refers to an explicit desire of the journal to include articles not based on novelty. This reflects the implicit mission of some journals to publish only novel journals, and not simply replications of previous work. Publishing replications of previous work and their results to some degree supports independent confirmation of the previous results, and also to a certain extent, addresses the file drawer problem ^9^.
- The category *Registered reports* refers to prospective peer review, i.e. evaluation of a manuscript submitted to a journal prior to data collection and/or data analysis. Registered reports have recently gained significant traction, with a few high-profile journals, including Nature Human Behaviour, Nature Communications, Scientific Reports and PLOS Biology, accepting this article that ‘frontloads’ peer review.
- *Open-science badges* refers to the use of so-called badges, which are awarded if a paper adheres to specific standards, thereby providing an incentive for promoting transparency and openness ^10^.

In summary, the TOP Factor covers major dimensions of open science and provides a helpful and standardised tool that allows to compare between journals or fields the extent to which they encourage or require adherence to open science principles.

## Methods

### TOP Factor

The TOP Factor (Transparency and Openness Factor; see extended data ^11^) is a quantitative score summarising the presence, requirement, and enforcement of transparent and open science practices in journals. It includes a total of ten sub-scales, of which nine score 0–3, and one scores 0–2, thereby resulting in a maximal summed score of 29. Higher values indicate a higher degree of adherence to the TOP practices.

### Journal identification

Journals to be included in the rating were identified using a hybrid pre-registered strategy ^12^:

- *Primary strategy.* Relevant journals were identified using search on the Web of Science Master Journal List (WoS MJL). The search terms, entered in separate searches, were:
  - “sleep”
  - “chronobiology”
  - “circadian”
  - “biological rhythms”
  - “dream”
- *Secondary strategies.* In addition to the primary search strategy, we used two supportive secondary strategies to identify relevant journals that may have been missed in the primary strategy:
  - “Own domain-relevant expertise in sleep and chronobiology;
  - “Informal consultation with a senior researcher with >25 years of experience in the field.
- *Validation.* The search results were merged, and duplicates were removed. We validated our search strategy by confirming that all journals listed in a recent publication on sleep research journals ^13^ were identified using this strategy. We validated our search strategy by confirming that the above search terms produce the same list of journals in MEDLINE ( https://www.ncbi.nlm.nih.gov/nlmcatalog?term=currentlyindexed%5BAll%20Fields%5D%20AND%20currentlyindexedelectronic%5BAll%20Fields%5D&cmd=DetailsSearch).

In addition to this strategy, we found two additional journals via searching the list of TOP signatories ( https://www.cos.io/initiatives/top-guidelines) for “sleep”, “chronobiology”, “circadian”, “biological rhythms” and “dream”, and one through a search in the National Library of Medicine (NLM).

### Journal meta-data extraction

We extracted the 2018 Journal Impact Factor (JIF) and the 5-year Journal Impact Factor from the *Clarivate Analytics InCites* platform. We obtained the NLM ID using search on the NLM data base, from which we also extracted the MEDLINE indexing status and the first year of publication. Information regarding support by scientific or professional societies extracted from both the NLM entry, and the journal website.

### Journal guidelines extraction

We consulted the journal websites for author guidelines. Where possible, we archived journal guidelines either locally, or on the Internet Wayback Machine.

### Scoring and conflict resolution

Three scorers (authors of this study, M.S., M.H.S, and C.B.) independently assessed the 27 identified journals’ TOP Factors in a total of 270 individual ratings (27 journals × 10 rating categories). In a first pass, the three scorers agreed in 77.7% of all ratings (210 out of 270 ratings; see underlying data ^11^). We then discussed and resolved major sources of discrepancy (e.g., we agreed that a clinical trial registration counted as preregistration), resolved some per-item disagreements and rescored the categories “Data citation” (initial disagreement rate: 13/27), “Reporting guidelines” (initial disagreement rate: 13/27) and “Study pre-registration” (initial disagreement rate: 19/27, see above) independently in a second pass (see underlying data ^11^). We report Fleiss’ kappa ^14^, intraclass correlation (ICC, calculated using the R package “irr”), and the disagreement rate for each item before resolving discrepancies. At the end of this second pass, we again entered a consensus process, by the end of which all ratings agreed. All scoring was completed between mid-May and mid-June 2020. Results are reported in Table 2.

## Results

### Journal identification

Through our search strategy, we identified a total of 28 journals. The 2018 JIF was available for 15 out of 28 journals (53.6%), and the 5-year JIF was available for all of these 15 except one (14 out of 28; 50%). Out of these 28 journals 11 out of 28, 39.2% of journals were not at present supported by a society. Three journals accepted submissions in a language other than English. Through our hybrid search strategy, we also found six additional journals (PLOS Neglected Tropical Diseases, International Journal of Gerontology, Lung India, Annals of Thoracic Medicine, Chronic Respiratory Disease, and Frontiers in Psychiatry), which we did not further consider, as they did not focus on sleep research or chronobiology, or were general purpose journals. One journal, *Sleep Medicine Reviews*, did not have any public author guidelines available, as it is an invite-only journal, and was therefore excluded, yielding a total number of 27 journals.

### Low explicit implementation of transparency and openness in sleep research and chronobiology journals

Across the 27 journals we examined, we find a total median TOP Factor of 3 (25 ^th^ percentile 1, 75 ^th^ percentile 4, minimum 0, maximum 9, IQR 3) out of a maximum of 29 points ( Table 1 and Table 2). The three journals scoring highest on the TOP were *Clocks & Sleep* (9), *Sleep Science and Practice* (7), and *Sleep and Vigilance* (6). Interestingly, these three journals were founded no earlier than 2017.

**Table 1.** Overview of included journals, sorted by 5-year impact factor. Data included are up to date as of May 2020.

Journal	ISSN / eISSN	NLM ID	Indexed in MEDLINE?	First year published	Publisher	Supporting Society	2018 Journal Impact Factor	5 Year Impact Factor
JOURNAL OF PINEAL RESEARCH	0742-3098 / 1600-079X	8504412	Yes	1984	Wiley	n/a	15.221	12.197
SLEEP MEDICINE REVIEWS	1087-0792 / 1532-2955	9804678	Yes	1997	Elsevier	n/a	10.517	10.255
SLEEP	0161-8105 / 1550-9109	7809084	Yes	1978	Oxford University Press	Sleep Research Society	4.571	5.588
JOURNAL OF SLEEP RESEARCH	0962-1105 / 1365-2869	9214441	Yes	1992	Wiley	European Sleep Research Society	3.432	3.951
SLEEP MEDICINE	1389-9457 / 1878-5506	100898759	Yes	2000	Elsevier	World Sleep Society and International Pediatric Sleep Association	3.36	3.934
JOURNAL OF CLINICAL SLEEP MEDICINE	1550-9389 / 1550-9397	101231977	Yes	2005	American Academy of Sleep Medicine	American Academy of Sleep Medicine	3.456	3.855
JOURNAL OF BIOLOGICAL RHYTHMS	0748-7304 / 1552-4531	8700115	Yes	1986	Sage	Society for Research on Biological Rhythms	2.473	3.349
BEHAVIORAL SLEEP MEDICINE	1540-2002 / 1540-2010	101149327	Yes	2003	Routledge Journals	Society of Behavioral Sleep Medicine	3.171	3.162
CHRONOBIOLOGY INTERNATIONAL	0742-0528 / 1525-6073	8501362	Yes	1984	Taylor & Francis	International Society for Chronobiology	2.562	2.998
SLEEP AND BREATHING	1520-9512 / 1522-1709	9804161	Yes	1997	Springer	Australasian Academy of Dental Sleep Medicine (AustADSM), the European Academy of Dental Sleep Medicine (EADSM), the Japanese Academy of Dental Sleep Medicine (JADSM), and Korean Academy of Dental Sleep Medicine (KADSM)	2.326	2.413
CRANIO-THE JOURNAL OF CRANIOMANDIBULAR & SLEEP PRACTICE	0886-9634 / 2151-0903	8609491	Yes	1983	Taylor & Francis	Alliance of TMD Organizations, American Academy of Craniofacial Pain (AACP), Tennessee C.R.A.N.I.O., Nederlandse Vereniging voor Gnathologie en Prothetische Tandheelkunde (NVGPT)	1.144	1.118
DREAMING	1053-0797 / 1573-3351	9111382	No	1991	American Psychological Association	International Association for the Study of Dreams	0.939	0.965
SLEEP AND BIOLOGICAL RHYTHMS	1446-9235 / 1479-8425	101199488	No	2003	Springer	Japanese Society of Sleep Research, Asian Sleep Research Society	0.752	0.954
BIOLOGICAL RHYTHM RESEARCH	0929-1016 / 1744-4179	9431857	No	1970	Taylor & Francis	n/a	0.773	0.691
SLEEP SCIENCE	1984-0659 / 1984-0063	101598477	No	2008	Brazilian Association of Sleep	Latin American Federation of Sleep Societies (FLASS - Federación Latinoamericana de Sociedades de Sueño)	n/a	n/a
CANADIAN JOURNAL OF RESPIRATORY CRITICAL CARE AND SLEEP MEDICINE	2474-5332 / 2474-5340	101740140	No	2017	Taylor & Francis	Canadian Thoracic Society	n/a	n/a
SLEEP HEALTH	2352-7218 / 2352-7226	101656808	Yes	2015	Elsevier	National Sleep Foundation	n/a	n/a
JOURNAL OF CIRCADIAN RHYTHMS	1740-3391	101200389	No	2003	Ubiquity Press	n/a	n/a	n/a
SOMNOLOGIE	1432-9123 / 1439-054X	9809663	No	1997	Springer	Deutschen Gesellschaft für Schlafforschung und Schlafmedizin (DGSM), Österreichischen Gesellschaft für Schlafmedizin und Schlafforschung (ÖGSM), Schweizerischen Gesellschaft für Schlafforschung, Schlafmedizin und Chronobiologie (SGSSC)	n/a	n/a
SLEEP MEDICINE: X	2590-1427	n/a	No	2019	Elsevier	n/a	n/a	n/a
CLOCKS & SLEEP	2624-5175	101736579	No	2019	MDPI	Australasian Chronobiology Society, Society for Light Treatment and Biological Rhythms, Swiss Society of Sleep Research, Sleep Medicine and Chronobiology	n/a	n/a
SLEEP SCIENCE AND PRACTICE	2398-2683	101739182	No	2017	Springer	n/a	n/a	n/a
CURRENT SLEEP MEDICINE REPORTS	2198-6401	101649332	No	2015	Springer	n/a	n/a	n/a
JOURNAL OF TURKISH SLEEP MEDICINE-TURK UYKU TIBBI DERGISI	2148-1504	n/a	No	2014	Galenos	Turkish Association of Sleep Medicine	n/a	n/a
NATURE AND SCIENCE OF SLEEP	1179-1608	101537767	No	2009	Dove Medical Press	n/a	3.054	n/a
NEUROBIOLOGY OF SLEEP AND CIRCADIAN RHYTHMS	2451-9944	101690253	No	2016	Elsevier	n/a	n/a	n/a
SLEEP AND VIGILANCE	2510-2265	101712170	No	2017	Springer	n/a	na	n/a
SLEEP MEDICINE CLINICS	1556-407X / 1556-4088	101271531	Yes	2006	Elsevier	n/a	n/a	n/a

**Table 2.** TOP ratings of included journals, sorted alphabetically by journal name.

Journal	TOP signatory status (yes, no)	TOP Factor: Data citation (0, 1, 2, or 3)	TOP Factor: Data transparency (0, 1, 2, or 3)	TOP Factor: Analytical code transparency (0, 1, 2, or 3)	TOP Factor: Materials Transparency (0, 1, 2, or 3)	TOP Factor: Reporting guidelines (0, 1, 2, or 3)	TOP Factor: Study prereg (0, 1, 2, or 3)	TOP Factor: Analysis prereg (0, 1, 2, or 3)	TOP Factor: Replication (0, 1, 2, or 3)	TOP Factor: Publication Bias (0, 1, 2, or 3)	TOP Factor: Open science badges (0, 1, or 2)	Total TOP
BEHAVIORAL SLEEP MEDICINE	No	2	1	0	0	0	0	0	0	0	0	**3**
BIOLOGICAL RHYTHM RESEARCH	No	2	1	0	0	0	1	0	0	0	0	**4**
CANADIAN JOURNAL OF RESPIRATORY CRITICAL CARE AND SLEEP MEDICINE	No	2	1	0	0	0	1	0	0	0	0	**4**
CHRONOBIOLOGY INTERNATIONAL	No	2	1	0	0	0	1	0	0	0	0	**4**
CLOCKS & SLEEP	No	2	2	0	2	1	1	1	0	0	0	**9**
CRANIO-THE JOURNAL OF CRANIOMANDIBULAR & SLEEP PRACTICE	No	2	1	0	0	0	1	0	0	0	0	**4**
CURRENT SLEEP MEDICINE REPORTS	Yes	0	1	1	1	0	1	0	0	0	0	**4**
DREAMING	No	0	0	0	0	0	0	0	0	0	0	**0**
JOURNAL OF BIOLOGICAL RHYTHMS	No	0	0	0	0	1	1	0	0	0	0	**2**
JOURNAL OF CIRCADIAN RHYTHMS	Yes	0	0	0	0	0	0	0	0	0	0	**0**
JOURNAL OF CLINICAL SLEEP MEDICINE	No	0	0	0	0	0	1	0	0	0	0	**1**
JOURNAL OF PINEAL RESEARCH	No	0	0	0	0	1	1	0	0	0	0	**2**
JOURNAL OF SLEEP RESEARCH	No	0	0	0	0	0	0	0	0	0	0	**0**
JOURNAL OF TURKISH SLEEP MEDICINE-TURK UYKU TIBBI DERGISI	No	0	0	0	0	2	1	0	0	0	0	**3**
NATURE AND SCIENCE OF SLEEP	No	0	0	0	0	1	1	0	0	0	0	**2**
NEUROBIOLOGY OF SLEEP AND CIRCADIAN RHYTHMS	Yes	1	0	0	0	1	1	0	0	0	0	**3**
SLEEP	No	0	0	0	0	1	1	0	0	0	0	**2**
SLEEP AND BIOLOGICAL RHYTHMS	Yes	0	0	0	0	0	1	0	0	0	0	**1**
SLEEP AND BREATHING	Yes	0	1	1	1	1	1	0	0	0	0	**5**
SLEEP AND VIGILANCE	Yes	1	1	1	1	1	1	0	0	0	0	**6**
SLEEP HEALTH	Yes	1	0	0	0	0	0	0	0	0	0	**1**
SLEEP MEDICINE	Yes	1	0	0	0	1	1	0	0	0	0	**3**
SLEEP MEDICINE CLINICS	No	0	0	0	0	0	0	0	0	0	0	**0**
SLEEP MEDICINE REVIEWS	No	0	0	0	0	0	0	0	0	0	0	**0**
SLEEP MEDICINE: X	No	1	0	0	0	1	1	0	0	0	0	**3**
SLEEP SCIENCE	No	0	0	0	0	0	1	0	0	0	0	**1**
SLEEP SCIENCE AND PRACTICE	No	2	2	0	2	1	0	0	0	0	0	**7**
SOMNOLOGIE	No	0	0	0	0	0	0	0	0	0	0	**0**
***Median***		0	0	0	0	0	1	0	0	0	0	**3**
***Q1***		0	0	0	0	0	0	0	0	0	0	**1**
***Q3***		1.5	1	0	0	1	1	0	0	0	0	**4**
***IQR***		1.5	1	0	0	1	1	0	0	0	0	**3**
***Minimum***		0	0	0	0	0	0	0	0	0	0	**0**
***Maximum***		2	2	1	2	2	1	1	0	0	0	**9**
Fleiss kappa’		1.00	0.70	0.68	0.76	1.00	0.94	0.49	-0.01	-0.01	NA	
ICC		1.00	0.79	0.69	0.71	1.00	0.94	0.50	0.00	0.00	NA	
# agreement		27	21	24	24	27	26	26	26	26	27	
% agreement		100.00	77.78	88.89	88.89	100.00	96.30	96.30	96.30	96.30	100.00	

To determine if journal prestige is correlated with TOP factor, we examined the correlation between IF and 5-year IF with Top Factor, finding no evidence for a significant rank correlation between IF and TOP Factor (Spearman’s *ρ*=-0.1012, p=0.7306; n=14) or 5-year IF and TOP Factor (Spearman's *ρ*=0.1389, p= 0.6509; n=13).

## Discussion

### Low uptake of transparent and openness principles

Our results, which focus on sleep research and chronobiology journals exclusively, are comparable to data on the uptake of the TOP guidelines across other disciplines. In 412 journals included in the Center for Open Science data base ( https://osf.io/qatkz/, Version: 19, 10 November 2020), the median TOP Factor is 2 (25th percentile 0, 75th percentile 8, minimum 0, maximum 27, IQR 8). Our subfield data are also comparable to a recent study examining transparency and openness in the field of pain research, which found a median TOP Factor of 3.5 (IQR 2.8) ^4, 5^.

As the TOP Factor was only launched in February 2020, we see the low transparency and openness scores in sleep research and chronobiology journals as an opportunity to revisit how we do science, and how we report it. Importantly, while the TOP Factor is a useful instrument to assess transparency and openness at the journal level, it is clear that science policy makers, scientific and learned societies, funders, and research institutions play a key role in incentivizing open science.

### Lack of a standard specification for journal guidelines

Across the 28 journals we examined, author guidelines varied widely in their accuracy, detail, and organisation of information. Many journals appeared to follow standard publisher guidelines, with very little or no modifications for the specific journal and often even referred to the publisher guidelines for further information. An additional challenge comes from the fact that the public-facing journal guidelines are not fully indicative of the process that the journal will implement, as further guidelines or requirements may be hidden in the submission system, or in correspondence with the journal during peer review or after acceptance of the article.

For example, it is unclear to what extent a rule will be enforced in the submission process when the guidelines say that authors ‘will be asked to’ do something. Fundamentally, this unseen information may limit the extent to which public author guidelines are truly reflective of the enforcement of transparency and openness principles in a given journal. In one instance, the editorial celebrating the inaugural issue of the journal stated that it welcomes Registered Reports, but at present, the author guidelines do not explicitly state this ^15^. Unless one was to consult this additional information, it would remain unknown to the author. One way to improve transparency and openness may be to devise a standard, machine- *and* human-readable specification schema for submission guidelines, reflecting the categories in the TOP Factor.

### Ambiguity in transparency and openness standards

There can be large ambiguity in the extent that a journal implements specific transparency and openness standards. Take, for example, the category “Study pre-registration”. There are four levels in this category: Level 0: *Journal says nothing*; Level 1: *Articles will state if work was preregistered*; Level 2: *Article states whether work was preregistered and, if so, journal verifies adherence to preregistered plan*; Level 3: *Journal requires that confirmatory or inferential research must be preregistered*. According to the TOP Guidelines (v1.0.1), Level 1 is satisfied if the research was registered in an independent, institutional registry, specifying “study design, variables, and treatment conditions prior to conducting the research”, leaving the level of detail open and rendering scorings ambiguous.

Indeed, there is a debate and confusion regarding the use of the terms *registration* vs. *pre-registration* ^16^. While the registration of a clinical trial in a trial registry such as clinicaltrials.gov can be relatively lightweight and contain only minimal details, a pre-registration (as used in the open science community) typically refers to the prospective specification of concrete study details, including methodology, sample size, and analysis plan prior to data collection in registries such as *AsPredicted.org* ( https://www.aspredicted.org/) or *OSF Preregistration* ( https://osf.io/prereg/) ^17^.

In more detail, the registration of a clinical trial in a registry such as clinicaltrials.gov on the one hand, and the pre-registration of analysis procedures and hypotheses prior to conducting the research on the other hand, mostly serve fundamentally different purposes, which is reflected in their nature too. First, clinical studies, which have not been registered, are impossible to publish in respected journals rendering the process a necessity rather than a self-imposed step to improve scientific transparency. Generally, when authors register a clinical trial (e.g. on the German Clinical Trials Register ^18^), they have to provide a short description of the trial, name the study goals, describe the intervention, name the primary endpoints, inclusion and exclusion criteria, the final sample size (without rationale), and the sponsor. Clearly, although the degree of detail is of course also subject to variation among pre-registered studies, the required level of detail for registering clinical studies is rather low, with accountability consequently likewise being very low. In some legislations (such as Switzerland), the submission of ethics application as a clinical trial (which is required for some studies that modify sleep schedules), by default deposits the study in the (Swiss) clinical trial registry ^19^.

Further developments of the TOP guidelines should therefore reflect the extent to which something has been preregistered, possibly also including at which time point during the scientific process the registration has taken place. Likewise, journals should be clear about what level of preregistration they expect.

### Linguistic details: When is ‘should’ mandatory?

The author guidelines also differed in the degree they used language to specify requirements. For example, many journals “encouraged” authors to do something, but the use of this term basically carries no power – you may also just ignore it. The use of the verb “should” may be intended to signal mandatory requirements, but it leaves the possibility of ignoring the requirement. Likewise, journals that “ask authors to do something” may still allow exceptions. This may not only be favourable for authors, who do not comply with the requirements, but also allows editors to treat some submissions different from others.

Moving forward, journals should state what aspects specified in guidelines are recommendations, what are requirements, and what the consequences for not meeting requirements are. To promote open science culture, it is clear that ‘hard’ requirements will need to replace ‘soft’ encouragement to promote the uptake of transparent and open research principles. Many transparent and open science principles require time and effort unless planned in advance. Many researchers are may not be convinced of the tangible benefits of specific open science practices. If, in addition to this, the reward is too low or non-existent, or there are no tangible negative consequences, even diligent scientists become a bit lazy, or simply prioritise non-optional tasks. Importantly, journal guidelines should frame requirements positively, and highlight the benefits of specific open-science practices, including discoverability of data and preventing the file-drawer effect.

### Open review as an additional open science dimension

Some journals, including eLife, PLOS, and Clocks & Sleep, now offer publishing of the pre-publication peer-review, with the possibility of naming the reviewers (if they agree). Publishing the review report and disclosing the reviewer identity are independent aspects of the broader ‘open review’ concept, as it is possible to disclose the reviewer identity to the authors as part of the journal submission and publication process without publishing it as part of the article, and it is also possible to publish the review report in anonymised form. There may of course be negative effects of disclosing the identity of reviewers, and potential reviewers may self-censor or fear retaliation if their names are known. Publishing the review reports does not only make the journey of an article from submission to publication transparent, laying open the shaping of an article through the peer review process. It can also curtail unreasonable requests during peer review and may encourage reviewers to provide constructive feedback oriented towards the best scientific outcome. We therefore encourage to include *Open review* as an additional category in future developments of the TOP guidelines, including the previously described shape that open peer review can take.

### The TOP Factor as a metric

Quantitative metrics can be subjected to being ‘gamed’, i.e., manipulated in such a way that they no longer correspond to corresponding to the original objective of the metric ^20, 21^. As principles of open science are becoming more important in hiring and funding decisions, the TOP Factor may similarly be subject to being ‘gamed’, with ‘openwashing’, i.e. the possible practice of superficially claiming adherence to open-science principles without ‘true’ engagement with them, being an immediate concern ^22^. Ultimately, whether or not this is a key driver will only be available for study retrospectively.

While the TOP Factor represents a first an important step in understanding the uptake of open and reproducible science systematically, it is important to note that some researchers may not be able to engage with open and reproducible science, due to institutional requirements, not least due to the effort required to implement them. Furthermore, there may be privacy or intellectual property concerns that could prevent the release of data, or the release of code pipelines. It is also conceivable that journals may not see open and reproducible practices as ‘nice to have’ but not ‘must have’ features or may think that they place a burden on the authors, thereby making the journal less attractive. This highlights the complexity of translating concepts of open, transparent and reproducible science into practice.

We further want to highlight the need to consider open science not as one ‘movement’, but as a set of practices, principles and policies from a wide and diverse “buffet” of options ^23^. The TOP Factor represents but one way of considering the extent to which one aspect of the research ecosystem – journals – can be summarised.

## Conclusion

In a comprehensive analysis of the author guidelines for 27 sleep research and chronobiology journals, we have found low evidence for explicit implementation of open and transparent science principles as assessed by the TOP Factor. We therefore encourage journals to make their requirements more explicit. Furthermore, to promote the recent developments in open, transparent and reproducible, journals should provide incentives for following open science practices.

## Data availability

### Underlying data

Open Science Framework: Transparency and open science principles in reporting guidelines in sleep research and chronobiology journals – Underlying and extended data. http://www.doi.org/10.17605/OSF.IO/KTMBH

This project contains the following underlying data:

- SupplementaryInformation_S1.xlsx (Intermediate scoring sheet, prior to resolving of reviewer disagreement and re-rating)
- SupplementaryInformation_S2.xlsx (Final scoring sheet)

### Extended data

Open Science Framework: Transparency and open science principles in reporting guidelines in sleep research and chronobiology journals – Underlying and extended data. http://www.doi.org/10.17605/OSF.IO/KTMBH this project contains the following extended data

- SupplementaryInformation_S3.pdf (TOP Factor scoring rubric)

Data are available under the terms of the Creative Commons Attribution 4.0 International license (CC-BY 4.0).

## Pre-print

A previous version of this article is available from bioRxiv: http://www.doi.org/10.1101/2020.06.26.172940 ^24^
