# Supplementary material for: Principles of open, transparent and reproducible science in author guidelines of sleep research and chronobiology journals
Source: Wellcome Open Res. 2021 Feb 26;5:172. Originally published 2020 Jul 20. [Version 2] doi: 10.12688/wellcomeopenres.16111.2 (PMC7941095; doi:10.12688/wellcomeopenres.16111.2)
Supplement: Peer review history

# Peer review: Reviewer response for version 2

**DOI:** 10.21956/wellcomeopenres.18320.r42864

**Published:** 2021-04-08

**Authors:** Gordon Feld, David Morgan, Juliane Tkotz

**Affiliations:** 1 Department of Clinical Psychology, Central Institute of Mental Health, Medical Faculty Mannheim, University of Heidelberg, Mannheim, Germany

I have no further comments.

Is the work clearly and accurately presented and does it cite the current literature?

Yes

If applicable, is the statistical analysis and its interpretation appropriate?

Yes

Are all the source data underlying the results available to ensure full reproducibility?

Yes

Is the study design appropriate and is the work technically sound?

Yes

Are the conclusions drawn adequately supported by the results?

Yes

Are sufficient details of methods and analysis provided to allow replication by others?

Yes

Reviewer Expertise:

Sleep and Memory

We confirm that we have read this submission and believe that we have an appropriate level of expertise to confirm that it is of an acceptable scientific standard.

# Peer review: Reviewer response for version 2

**DOI:** 10.21956/wellcomeopenres.18320.r42865

**Published:** 2021-03-08

**Authors:** Elizabeth Beryl Klerman

**Affiliations:** 1 Division of Sleep and Circadian Disorders, Department of Medicine, Brigham and Women's Hospital, Boston, MA, USA; 2 Massachusetts General Hospital, Boston, MA, USA

I have no further suggestions or comments.  I recommend publication.

Is the work clearly and accurately presented and does it cite the current literature?

Yes

If applicable, is the statistical analysis and its interpretation appropriate?

Not applicable

Are all the source data underlying the results available to ensure full reproducibility?

Yes

Is the study design appropriate and is the work technically sound?

Yes

Are the conclusions drawn adequately supported by the results?

Yes

Are sufficient details of methods and analysis provided to allow replication by others?

Yes

Reviewer Expertise:

NA

I confirm that I have read this submission and believe that I have an appropriate level of expertise to confirm that it is of an acceptable scientific standard.

# Peer review: Reviewer response for version 1

**DOI:** 10.21956/wellcomeopenres.17684.r39631

**Published:** 2020-09-01

**Authors:** Evan Mayo-Wilson

**Affiliations:** 1 Department of Epidemiology and Biostatistics, Indiana University School of Public Health-Bloomington, Bloomington, IN, USA

It is important to describe the extent to which journals support transparency so that we can help journals support greater transparency and openness through their policies and procedures.

 The TOP Factor is an appropriate framework to describe these issues, but it has some limitations. Notably, I would not describe TOP factor as an “instrument.” Like the TOP guidelines, the TOP factor includes normative standards for transparency. It does not provide structured questions to determine whether those standards have been met. Some aspects of the TOP guidelines are difficult to rate because the standards were not developed to be used as a rating instrument. Each of the TOP guidelines includes multiple components, and journal policy language can be unclear and contradictory. For example, this manuscript says that “ *Replication* refers to an explicit desire of the journal to include articles not based on novelty,” which is part of, but not the entirety of, a TOP standard designed to promote the publication of replication studies. It is unclear how the authors handled this and other compound standards/questions. The discussion also raises the problem of unclear/imprecise language like “should,” but the manuscript does not explain how the authors coded policies with unclear language.

 The methods used to assess agreement are very limited. Given the proportion of journal policies that were rated level 0, the total agreement should be extremely high. For example, it is concerning that the authors disagreed about 13/28 journals for one item, which suggests that the “instrument” is not reliable. At a minimum, the authors should report kappa and specific agreement (e.g., agreement for the categories), and they should consider the implications of poor reliability for efforts to measure and improve transparency and openness.

 The authors rated a small number of journals related to sleep research, a field in which I am not an expert. It seems possible that these journals would be representative of this specific field of research. Transparency is poor in many disciplines, but these specific results might have limited generalizability as well as limited internal validity.

Is the work clearly and accurately presented and does it cite the current literature?

Yes

If applicable, is the statistical analysis and its interpretation appropriate?

Partly

Are all the source data underlying the results available to ensure full reproducibility?

Yes

Is the study design appropriate and is the work technically sound?

Partly

Are the conclusions drawn adequately supported by the results?

Partly

Are sufficient details of methods and analysis provided to allow replication by others?

No

Reviewer Expertise:

Clinical trials, evidence synthesis, research transparency

I confirm that I have read this submission and believe that I have an appropriate level of expertise to confirm that it is of an acceptable scientific standard, however I have significant reservations, as outlined above.

# Response

**Published:** 2021-02-01

**Authors:** Manuel Spitschan

**Affiliations:** University of Oxford, UK

*We thank the author for their comments, and agree that there is probably room for improved in the methodology that we used. We have removed the description of the TOP Factor as an instrument in the abstract. We have also now reported kappa, ICC, and % agreement.*

# Peer review: Reviewer response for version 1

**DOI:** 10.21956/wellcomeopenres.17684.r39630

**Published:** 2020-08-03

**Authors:** Elizabeth Beryl Klerman

**Affiliations:** 1 Division of Sleep and Circadian Disorders, Department of Medicine, Brigham and Women's Hospital, Boston, MA, USA; 2 Massachusetts General Hospital, Boston, MA, USA

**Overall**

 This is an interesting article on an important topic. There is not sufficient discussion or rationale for some of the statements relating to the importance of this topic. The authors assume that transparency and open science principles are already accepted by all scientists/journal and that all aspects of these principles are equally important. More text about why these principles are important overall and for each principle as well as a recognition why a scientist or journal might not want to adhere to all principles would make this a more balanced report. Minor comment: some text is in the wrong area (e.g., methods related text in Results section).

**Specific**

- *Abstract:* need more justification for why adherence should be mandatory.
- *P2 Introduction, 3 ^rd^ paragraph:* Refs 4 and 5 are both in the same field of pain; that is not evidence of “publications in other fields” (plural).
- *P3 Introduction:* Please add text about why each principle or group of principles are important for science.
- P3 Methods: Reference 16 is to another version of this paper. It is not independent validation.
- *P3 Methods; bullet beginning “The search results were merged…”:* The Validation text should be under Validation bullet.
- *P4 Method, left panel:* What strategy was used to find the two additional journals?
- *P4 Methods. Journal meta-data extraction:* Information about what percent of journal were supported by a society and what percent were in language that was not English belongs in Results (unless those criteria are exclusionary).
- *P4 Methods. Journal guidelines extraction:* Information about no public author guidelines belongs in Results.
- *P4 Methods:*The date of registration of this study in a registry and the name of the registry is not given. When I looked at the registry (from a link in this paper), this study was registered on July 13, after the research had begun. Is that correct? Did the authors not follow TOP principles in this paper?
- *P4 Results:* Begin with the number of journals found.
- *P4 Results. Last line of left panel: *The text beginning “our Results compare…” belongs in Discussion.
- *P4 Results “Lack of a standard specification for journal guidelines”:* It is not clear why the information in the first two sentences should be considered a problem. Most of the rest of the paragraph belongs in the Discussion.
- *P4 Discussion:* Please give a few examples of such registries in the main text or in Supplemental materials. Readers might not be aware of the many ones available.
- *P9 Discussion: *Another reason besides laziness why are researcher might not want to complete optional requirements is the many other tasks they might need to complete that are not optional.
- *P9 Discussion:* Please acknowledge the potential negative effect of having the name of a reviewer revealed. I personally feel like I can be less honest when my name is known to the authors because of potential effects on our inter-personal relationship(s). I don’t think I am unreasonable in my requests – and the author can always refuse to do those requests.
- *P9 Discussion:* Please also discuss/acknowledge why a researcher or journal might not want to fully comply with all TOP factors.
- *S1 and S1:* What were the criteria for excluding the journals on the Removed tab?
- *S3 PDF:* Please label what it is within the document.

Is the work clearly and accurately presented and does it cite the current literature?

Yes

If applicable, is the statistical analysis and its interpretation appropriate?

Not applicable

Are all the source data underlying the results available to ensure full reproducibility?

Yes

Is the study design appropriate and is the work technically sound?

Yes

Are the conclusions drawn adequately supported by the results?

Yes

Are sufficient details of methods and analysis provided to allow replication by others?

Yes

Reviewer Expertise:

sleep research and chronobiology

I confirm that I have read this submission and believe that I have an appropriate level of expertise to confirm that it is of an acceptable scientific standard, however I have significant reservations, as outlined above.

# Response

**Published:** 2021-02-01

**Authors:** Manuel Spitschan

**Affiliations:** University of Oxford, UK

***Reviewer 2***

*This is an interesting article on an important topic. There is not sufficient discussion or rationale for some of the statements relating to the importance of this topic. The authors assume that transparency and open science principles are already accepted by all scientists/journal and that all aspects of these principles are equally important. More text about why these principles are important overall and for each principle as well as a recognition why a scientist or journal might not want to adhere to all principles would make this a more balanced report. Minor comment: some text is in the wrong area (e.g., methods related text in Results section).*

**

***Specific***

- *Abstract: need more justification for why adherence should be mandatory.*
*  *
- *P2 Introduction, 3 ^rd^ paragraph: Refs 4 and 5 are both in the same field of pain; that is not evidence of “publications in other fields” (plural).*
*  *
- *P3 Introduction: Please add text about why each principle or group of principles are important for science.*
*  *
- *P3 Methods: Reference 16 is to another version of this paper. It is not independent validation.*
*  *
- *P3 Methods; bullet beginning “The search results were merged…”: The Validation text should be under Validation bullet.*
*  *
- *P4 Method, left panel: What strategy was used to find the two additional journals?*
*  *
- *P4 Methods. Journal meta-data extraction: Information about what percent of journal were supported by a society and what percent were in language that was not English belongs in Results (unless those criteria are exclusionary).*
*  *
- *P4 Methods. Journal guidelines extraction: Information about no public author guidelines belongs in Results.*
*  *
- *P4 Methods:The date of registration of this study in a registry and the name of the registry is not given. When I looked at the registry (from a link in this paper), this study was registered on July 13, after the research had begun. Is that correct? Did the authors not follow TOP principles in this paper?*
*  *
- *P4 Results: Begin with the number of journals found.*
*  *
- *P4 Results. Last line of left panel: The text beginning “our Results compare…” belongs in Discussion.*
*  *
- *P4 Results “Lack of a standard specification for journal guidelines”: It is not clear why the information in the first two sentences should be considered a problem. Most of the rest of the paragraph belongs in the Discussion.*
*  *
- *P4 Discussion: Please give a few examples of such registries in the main text or in Supplemental materials. Readers might not be aware of the many ones available.*
*  *
- *P9 Discussion: Another reason besides laziness why are researcher might not want to complete optional requirements is the many other tasks they might need to complete that are not optional.*
*  *
- *P9 Discussion: Please acknowledge the potential negative effect of having the name of a reviewer revealed. I personally feel like I can be less honest when my name is known to the authors because of potential effects on our inter-personal relationship(s). I don’t think I am unreasonable in my requests – and the author can always refuse to do those requests.*
*  *
- *P9 Discussion: Please also discuss/acknowledge why a researcher or journal might not want to fully comply with all TOP factors.*
*  *
- *S1 and S1: What were the criteria for excluding the journals on the Removed tab?*
*  *
- *S3 PDF: Please label what it is within the document.*

We thank the reviewer for the comments.

*Abstract: need more justification for why adherence should be mandatory.*

 We have now revised the abstract, and have removed the reference to mandatory adherence. Ultimately, we do not think that forcing authors to adhere to specific guidelines will lead to culture change.

*P2 Introduction, 3rd paragraph: Refs 4 and 5 are both in the same field of pain; that is not evidence of “publications in other fields” (plural).*

 We have now revised this sentence to specifically refer to pain research.

*P3 Introduction: Please add text about why each principle or group of principles are important for science.*

 We now added more text describing why each of these principles may be important for science, giving the reader more context.

*P3 Methods: Reference 16 is to another version of this paper. It is not independent validation.*

 We now refer to Reference 10, a publication in the *Journal of Sleep Research* (Marshall NS, Hoyos CM: Impact factor rankings for sleep research journals between 2005 and 2018. J Sleep Res. 2020;e13015. 10.1111/jsr.13015), which examined the Impact Factor of sleep research journals.

*P3 Methods; bullet beginning “The search results were merged…”: The Validation text should be under Validation bullet.*

 We have now moved this to the Validation section.

*P4 Method, left panel: What strategy was used to find the two additional journals?*

 We have now included this information.

**

*P4 Methods. Journal meta-data extraction: Information about what percent of journal were supported by a society and what percent were in language that was not English belongs in Results (unless those criteria are exclusionary).*

 We have now moved this to the Results section.

*P4 Methods. Journal guidelines extraction: Information about no public author guidelines belongs in Results.*

**

 We have now moved this to the Results section.

*P4 Methods:The date of registration of this study in a registry and the name of the registry is not given. When I looked at the registry (from a link in this paper), this study was registered on July 13, after the research had begun. Is that correct? Did the authors not follow TOP principles in this paper?*

 The authors must have looked at the *Extended Dataset*, which we uploaded to the Open Science Framework with a registration date of 13 July 2020 ( https://doi.org/10.17605/OSF.IO/KTMBH), in line with recommendations from Wellcome Open Research. Our pre-registration of the work was registered on 14 May 2020 ( https://doi.org/10.17605/OSF.IO/QNSBM).

*P4 Results: Begin with the number of journals found.*

 We have now included this in the Results section.

*P4 Results. Last line of left panel: The text beginning “our Results compare…” belongs in Discussion.*

 We have now moved this to the Discussion section.

*P4 Results “Lack of a standard specification for journal guidelines”: It is not clear why the information in the first two sentences should be considered a problem. Most of the rest of the paragraph belongs in the Discussion.*

 This paragraph was moved to the discussion.

*P4 Discussion: Please give a few examples of such registries in the main text or in Supplemental materials. Readers might not be aware of the many ones available.*

 We have now added references to the two main pre-registration sites: AsPredicted.org and OSF Pregistration.

*P9 Discussion: Another reason besides laziness why are researcher might not want to complete optional requirements is the many other tasks they might need to complete that are not optional.*

 We have revised this to include the nuance that researchers may simply prioritise non-optional tasks.

*P9 Discussion: Please acknowledge the potential negative effect of having the name of a reviewer revealed. I personally feel like I can be less honest when my name is known to the authors because of potential effects on our inter-personal relationship(s). I don’t think I am unreasonable in my requests – and the author can always refuse to do those requests.*

 We have now revised this section to say:

 Some journals, including eLife, PLOS, and Clocks & Sleep, now offer publishing of the pre-publication peer-review, with the possibility of naming the reviewers (if they agree). Publishing the review report and disclosing the reviewer identity are independent aspects of the broader 'open review' concept, as it is possible to disclose the reviewer identity to the authors as part of the journal submission and publication process without publishing it as part of the article, and it is also possible to publish the review report in anonymised form. There may of course be negative effects of disclosing the identity of reviewers, and potential reviewers may self-censor or fear retaliation if their names are known. Publishing the review reports does not only make the journey of an article from submission to publication transparent, laying open the shaping of an article through the peer review process. It can also curtail unreasonable requests during peer review and may encourage reviewers to provide constructive feedback oriented towards the best scientific outcome. We therefore encourage to include *Open review *as an additional category in future developments of the TOP guidelines, including the previously described shape that open peer review can take.

*P9 Discussion: Please also discuss/acknowledge why a researcher or journal might not want to fully comply with all TOP factors.*

 We now address this in the discussion:

**

*S1 and S1: What were the criteria for excluding the journals on the Removed tab?*

 We have now stated these criteria in the Results section.

*S3 PDF: Please label what it is within the document.*

 This is included in the *Extended Data* section.

# Peer review: Reviewer response for version 1

**DOI:** 10.21956/wellcomeopenres.17684.r39633

**Published:** 2020-07-30

**Authors:** Gordon Feld, Juliane Tkotz, David Morgan

**Affiliations:** 1 Department of Clinical Psychology, Central Institute of Mental Health, Medical Faculty Mannheim, University of Heidelberg, Mannheim, Germany

The authors assess the implementation of open science practices in journal author guidelines in the field of sleep and chronobiology. The authors achieved this by calculating the Transparency and Openess (TOP) factor for 28 sleep and chronobiology journals. They find that overall, open science practices are poorly represented within sleep and chronobiology journals and call for more explicit open science guidelines, e.g. via replacing “soft encouragement” with “hard requirements”. The implementation of open science practices is an important matter and the manuscript helps to raise awareness that many publishers still do not encourage or require these practices. With that said, the present work only provides a limited overview for the field of sleep and chronobiology, without providing a goal post or a broader reference point (except for data from the field of pain research). I think a broader comparison across several fields would be more informative. However, given that few papers acknowledge open science issues in sleep research this paper is a breath of fresh air towards important discussion of such matters.

 Issues that might be addressed in the current manuscript are:

1. What I am missing is a reference point. The authors mention the TOP factor in pain research on page 4, but I would like to see some general information about the distribution of TOP factors and a descriptive comparison of where sleep journals stand relative to others in general, as e.g. done in this blog article:
https://www.natureindex.com/news-blog/top-factor-rates-journals-on-transparency-openness. After all, sleep research is not only published in sleep-related journals.
2. Maybe explicitly mention when the TOP factor was launched (Feb 2020), i.e. how much time there was to implement it. Of course, open science practices could (and should) have been implemented before the TOP factor, but given that the measure is around for a short period of time, it is reasonable to expect that journal requirements are not yet geared towards it.
3. Another point to discuss would be the potential misuse of the TOP factor, i.e. some form of “gamification” or “open washing”, where the TOP factor becomes the new impact factor ( https://go.nature.com/3gat5Vl). It may also be worth discussing whether the TOP factor is enough to encourage good science. Especially in sleep science there is a tendency to collect very small samples and as far as I can see there is no measurement for this included in the TOP factor (e.g., a priori power analyses with smallest effect size of interest). Also, funders and universities may play a much more important role in incentivizing open and reproducible science.
4. The authors collected data on the impact factor – it would be interesting to compare the general association between impact factor and TOP factor. However, the sample size (especially considering that the impact factor was only available for a subset of the journals) might be too small for a meaningful overview. Then it would be useful to nevertheless offer a scatter plot.
5. The authors rightly point out that journals should adopt hard requirements to encourage scientists to practice open science (page 9). This is then followed by a series of reasons why researchers have not adopted open science practices e.g., it costs time. It might be nice to remind the reader of the positive reasons to adopt open science practices (e.g., https://bit.ly/2X0nOrJ).
6. It is great that the authors have provided the data. However for the second excel file SuplementaryInformation_S2.xlsx, sheet "comparison", the syntax used does not appear to consistently generate the arrays of the rater ratings – might be worth checking this out to see what is going wrong. It might also be helpful to provide an aggregate sheet in that file as was the case in the SuplementaryInformation_S1.xlsx file.
7. The tables in the manuscript are a bit tedious to read. The authors could consider condensing them a bit. Ideally, the tables would be formatted to be publishable in portrait rather than landscape format. This may be achieved by choosing a smaller font and putting some of the labels into the table legend. In case the publisher intends to reformat these tables, this comment can largely be ignored.

Is the work clearly and accurately presented and does it cite the current literature?

Yes

If applicable, is the statistical analysis and its interpretation appropriate?

Yes

Are all the source data underlying the results available to ensure full reproducibility?

Yes

Is the study design appropriate and is the work technically sound?

Yes

Are the conclusions drawn adequately supported by the results?

Yes

Are sufficient details of methods and analysis provided to allow replication by others?

Yes

Reviewer Expertise:

Sleep and Memory

We confirm that we have read this submission and believe that we have an appropriate level of expertise to confirm that it is of an acceptable scientific standard, however we have significant reservations, as outlined above.

# Response

**Published:** 2021-02-01

**Authors:** Manuel Spitschan

**Affiliations:** University of Oxford, UK

**We thank the reviewers for their comments, which we think have greatly improved the manuscript. In revising this manuscript, we have revisited specific sections in the Methods and our calculations, some of which required corrections, which we have now made in the manuscript.**

**Reviewer 1**

 The authors assess the implementation of open science practices in journal author guidelines in the field of sleep and chronobiology. The authors achieved this by calculating the Transparency and Openess (TOP) factor for 28 sleep and chronobiology journals. They find that overall, open science practices are poorly represented within sleep and chronobiology journals and call for more explicit open science guidelines, e.g. via replacing “soft encouragement” with “hard requirements”. The implementation of open science practices is an important matter and the manuscript helps to raise awareness that many publishers still do not encourage or require these practices. With that said, the present work only provides a limited overview for the field of sleep and chronobiology, without providing a goal post or a broader reference point (except for data from the field of pain research). I think a broader comparison across several fields would be more informative. However, given that few papers acknowledge open science issues in sleep research this paper is a breath of fresh air towards important discussion of such matters.

 Issues that might be addressed in the current manuscript are:

1. What I am missing is a reference point. The authors mention the TOP factor in pain research on page 4, but I would like to see some general information about the distribution of TOP factors and a descriptive comparison of where sleep journals stand relative to others in general, as e.g. done in this blog article:
https://www.natureindex.com/news-blog/top-factor-rates-journals-on-transparency-openness. After all, sleep research is not only published in sleep-related journals.
2. Maybe explicitly mention when the TOP factor was launched (Feb 2020), i.e. how much time there was to implement it. Of course, open science practices could (and should) have been implemented before the TOP factor, but given that the measure is around for a short period of time, it is reasonable to expect that journal requirements are not yet geared towards it.
3. Another point to discuss would be the potential misuse of the TOP factor, i.e. some form of “gamification” or “open washing”, where the TOP factor becomes the new impact factor ( https://go.nature.com/3gat5Vl). It may also be worth discussing whether the TOP factor is enough to encourage good science. Especially in sleep science there is a tendency to collect very small samples and as far as I can see there is no measurement for this included in the TOP factor (e.g., a priori power analyses with smallest effect size of interest). Also, funders and universities may play a much more important role in incentivizing open and reproducible science.
4. The authors collected data on the impact factor – it would be interesting to compare the general association between impact factor and TOP factor. However, the sample size (especially considering that the impact factor was only available for a subset of the journals) might be too small for a meaningful overview. Then it would be useful to nevertheless offer a scatter plot.
5. The authors rightly point out that journals should adopt hard requirements to encourage scientists to practice open science (page 9). This is then followed by a series of reasons why researchers have not adopted open science practices e.g., it costs time. It might be nice to remind the reader of the positive reasons to adopt open science practices (e.g., https://bit.ly/2X0nOrJ).
6. It is great that the authors have provided the data. However for the second excel file SuplementaryInformation_S2.xlsx, sheet "comparison", the syntax used does not appear to consistently generate the arrays of the rater ratings – might be worth checking this out to see what is going wrong. It might also be helpful to provide an aggregate sheet in that file as was the case in the SuplementaryInformation_S1.xlsx file.
7. The tables in the manuscript are a bit tedious to read. The authors could consider condensing them a bit. Ideally, the tables would be formatted to be publishable in portrait rather than landscape format. This may be achieved by choosing a smaller font and putting some of the labels into the table legend. In case the publisher intends to reformat these tables, this comment can largely be ignored.

We thank the reviewers for their comments.

*What I am missing is a reference point. The authors mention the TOP factor in pain research on page 4, but I would like to see some general information about the distribution of TOP factors and a descriptive comparison of where sleep journals stand relative to others in general, as e.g. done in this blog article:*

*https://www.natureindex.com/news-blog/top-factor-rates-journals-on-transparency-openness* *. After all, sleep research is not only published in sleep-related journals.*

 We have now included a section comparing the TOP Factors we found with journals registered in the Center for Open Science data base:

 Our results, which focus on sleep research and chronobiology journals exclusively, are comparable to data on the uptake of the TOP guidelines across other disciplines. In 412 journals included in the Center for Open Science data base (https://osf.io/qatkz/, Version: 19, 10 November 2020), the median TOP Factor is 2 (25th percentile 0, 75th percentile 8, minimum 0, maximum 27, IQR 8).  Our subfield data are also comparable to a recent study examining transparency and openness in the field of pain research, which found a median TOP Factor of 3.5 (IQR 2.8) ^4^^, ^^5^ .

 This now serves as a useful reference for the comparison, indicating that the results found here are very much comparable with other journals as well.

*Maybe explicitly mention when the TOP factor was launched (Feb 2020), i.e. how much time there was to implement it. Of course, open science practices could (and should) have been implemented before the TOP factor, but given that the measure is around for a short period of time, it is reasonable to expect that journal requirements are not yet geared towards it.*

 Point 2: We have now addressed this in the introduction, stating that it was launched in February 2020, and also have added a section about this in the discussion. We write:

 As the TOP Factor was only launched in February 2020, we see the low transparency and openness scores in sleep research and chronobiology journals as an opportunity to revisit how we do science, and how we report it. Importantly, while the TOP Factor is a useful instrument to assess transparency and openness at the journal level, it is clear that science policy makers, scientific and learned societies, funders, and research institutions play a key role in incentivizing open science.

*Another point to discuss would be the potential misuse of the TOP factor, i.e. some form of “gamification” or “open washing”, where the TOP factor becomes the new impact factor (* *https://go.nature.com/3gat5Vl* *). It may also be worth discussing whether the TOP factor is enough to encourage good science. Especially in sleep science there is a tendency to collect very small samples and as far as I can see there is no measurement for this included in the TOP factor (e.g., a priori power analyses with smallest effect size of interest). Also, funders and universities may play a much more important role in incentivizing open and reproducible science.*

 We have now addressed this in the discussion, where we write:

**Gaming the TOP Factor?**

 Quantitative metrics can be subjected to being ‘gamed’, i.e., manipulated in such a way that they no longer correspond to corresponding to the original objective of the metric ^17, 18^. As principles of open science are becoming more important in hiring and funding decisions, the TOP Factor may similarly be subject to being ‘gamed’, with ‘openwashing’ being an immediate concern ^19^.  Ultimately, whether or not this is a key driver will only be available for study retrospectively.

**

*The authors collected data on the impact factor – it would be interesting to compare the general association between impact factor and TOP factor. However, the sample size (especially considering that the impact factor was only available for a subset of the journals) might be too small for a meaningful overview. Then it would be useful to nevertheless offer a scatter plot.*

 We now reported the rank correlation, but there is no convincing evidence. As the data are available in the tables, any interested readers will be able to follow up with their own assessments.

*The authors rightly point out that journals should adopt hard requirements to encourage scientists to practice open science (page 9). This is then followed by a series of reasons why researchers have not adopted open science practices e.g., it costs time. It might be nice to remind the reader of the positive reasons to adopt open science practices (e.g.,* *https://bit.ly/2X0nOrJ* *).*

**

 We have now revised this and highlighted the positive aspects, and also stated that journal guidelines should be framed positively.

*It is great that the authors have provided the data. However for the second excel file SuplementaryInformation_S2.xlsx, sheet "comparison", the syntax used does not appear to consistently generate the arrays of the rater ratings – might be worth checking this out to see what is going wrong. It might also be helpful to provide an aggregate sheet in that file as was the case in the SuplementaryInformation_S1.xlsx file.*

**

 We provide the second table only as a raw record of the intermediate data, and do not think that this will be useful for readers, but have included it for completeness. We would be happy to remove it.

*The tables in the manuscript are a bit tedious to read. The authors could consider condensing them a bit. Ideally, the tables would be formatted to be publishable in portrait rather than landscape format. This may be achieved by choosing a smaller font and putting some of the labels into the table legend. In case the publisher intends to reformat these tables, this comment can largely be ignored.*

 Point 7: The formatting of the tables is ultimately up to the publisher and we therefore will leave it to them.
